# Increased Levels of miR-15b-5p and miR-20b-5p in Pancreatic Ductal Adenocarcinoma with Hepatic Metastases

**DOI:** 10.3390/genes14081577

**Published:** 2023-08-02

**Authors:** Maria Dobre, Radu Cristian Poenaru, Andrei Marian Niculae, Catalina Vladut, Vlad Herlea, Elena Milanesi, Mihail Eugen Hinescu

**Affiliations:** 1 Victor Babes National Institute of Pathology, 050096 Bucharest, Romania; maria_dobre70@yahoo.com (M.D.); niculae_andrei92@yahoo.com (A.M.N.); mhinescu@yahoo.com (M.E.H.); 2 Faculty of Medicine, Carol Davila University of Medicine and Pharmacy, 050474 Bucharest, Romania; poenarucristian23@gmail.com (R.C.P.); drcatalinavladut@gmail.com (C.V.); 3 Department of Gastroenterology, “Prof. Dr. Agrippa Ionescu” Clinical Emergency Hospital, 011356 Bucharest, Romania; 4 Fundeni Clinical Institute, 022328 Bucharest, Romania; herlea2002@yahoo.com

**Keywords:** PDAC, hepatic metastases, miR-15b-5p, miR-20b-5p

## Abstract

Pancreatic ductal adenocarcinoma (PDAC) is one of the most aggressive and lethal forms of cancer. The symptoms appear in advanced stages, and diagnostic and prognostic tests for the early detection of PDAC and disease evolution are not available. The dysregulation of microRNAs (miRNAs) has been associated with cancer development and progression, and some miRNAs have been reported to promote specific metastasis. In this study we aimed to identify the miRNAs dysregulated in PDAC tumoral tissues and a subset of miRNAs associated with tumoral characteristics, mainly metastasis presence and site. For this, the expression of 84 miRNAs was evaluated by qPCR in 30 tumoral tissues and 16 samples of non-tumoral pancreatic tissues. The comparison revealed 32 dysregulated miRNAs (19 upregulated and 13 downregulated) in the PDAC group. Reactome pathway over-representation analysis revealed that these miRNAs are involved in several biological pathways, including “ESR-mediated signaling”, “PIP3 activates AKT signaling”, and “Regulation of PTEN”, among others. Moreover, our study identified an upregulation of miR-15b-5p and miR-20b-5p in the tumoral tissues of patients with hepatic metastasis, outlining these miRNAs as potential markers for hepatic metastasis. No significant difference in miRNA expression was observed in relation to anatomic location, lymphovascular invasion, lung metastasis, and the presence of diabetes.

## 1. Introduction

According to a recent GLOBOCAN study, pancreatic cancer had 495,773 new cases in 2020, meaning that it ranks fourteenth among the most prevalent malignancies. Moreover, it was the seventh most common cause of cancer fatalities, accounting for 466,003 deaths in 2020. These numbers represent 2.6% of all new cancer diagnoses and 4.7% of all cancer deaths, respectively [1]. In 2023, there will likely be 64,050 new cases of pancreatic cancer identified in the U.S., with 50,550 deaths anticipated as a result of the diagnosis. According to the American Cancer Society, the 5-year relative survival rates for localized, regional, distant, and all stages combined were 44%, 15%, 3%, and 12%, respectively [2]. Unfortunately, these survival rates continue to be low. This can partly be attributed to the fact that most diagnoses are made only at an advanced stage when patients present the initial symptoms. An analysis of the percentage of cases with localized, regional, or distant-stage disease for those who received a pancreatic cancer diagnosis in the United States between 2007 and 2011 revealed that more than 50% of patients have a metastatic disease, with only about 10% of pancreatic tumors being localized and resectable [3]. Over 85% of pancreatic malignancies are adenocarcinomas [3], which are most commonly found in the head of the pancreatic tissue [4]. During the last 10 years several risk factors involved in PDAC have been identified and classified as modifiable and not modifiable risk factors. The main modifiable risk factors related to pancreatic cancer are represented by lifestyle habits, including the consumption of tobacco and alcohol, a high body mass index, and obesity [5]. Regarding non-modifiable factors, a ten-fold increase in the risk of PDAC has been estimated in individuals with a family history of pancreatic cancer (at least three first-, second-, third-degree), hereditary pancreatitis, Peutz-Jeghers syndrome, and familial atypical multiple mole melanoma, whereas a moderate increase (5–10-fold increase) has been found in individuals with BRCA2 mutations, chronic pancreatitis, cystic fibrosis, and a family history of pancreatic cancer in two first-degree relatives [6].

According to a growing body of research, neoadjuvant chemotherapy is increasingly being used as a therapeutic option for patients with pancreatic cancer who may otherwise be candidates for surgery [7]. Conroy et al. demonstrated that, despite having a greater prevalence of severe side effects, adjuvant treatment with a modified FOLFIRINOX regimen (leucovorin, 5-fluorouracil, irinotecan and oxaliplatin) resulted in considerably longer survival times than gemcitabine among patients with resected pancreatic cancer [8]. A systematic review and meta-analysis published by Zhang and colleagues showed that nabpaclitaxel plus gemcitabine is the first-line therapy for patients with pancreatic ductal adenocarcinoma (PDAC) in its advanced stages, showing a median overall survival ranging from 6.9 months to 24.7 months, with a 1-year survival rate of 45.2% [9].

In terms of diagnostic, prognostic, and surveillance capabilities, CA19-9 is likely the biomarker that has undergone the most rigorous validation and investigation, and it is the only biomarker for the diagnosis and monitoring of PDAC that has received FDA approval [10]. However, it has been recently shown that the detection of soluble AXL receptor tyrosine kinase (sAXL) in plasma samples represents a novel biomarker for early-stage PDAC diagnosis and that its levels can more accurately discriminate PDAC from pancreatitis than CA19-9 [11].

Thorough studies have been carried out for the identification of new potential biomarkers to help in pancreatic cancer screening, diagnosis, and treatment. Investigations have been conducted into the detection of biomarkers in the pancreatic juice, cyst fluid, blood, bile, and urine, as well as on pancreatic tumoral tissue using genomic, transcriptomic, proteomic, and metabolomic approaches. However, all of these novel biomarkers are yet to be brought into routine clinical practice [12].

MicroRNAs (miRNAs) are non-coding small RNAs composed of 17–25 nucleotides that can regulate the expression of multiple genes in the post-transcriptional process. Since they play an important role in carcinogenesis, especially in the case of tumor-suppressive miRNAs and oncogenic miRNAs, they have been indicated as promising biomarkers for diagnosis [13], prognosis, and drug response prediction, as well as therapeutic targets and as marker s of metastatic disease [14].

Hepatic metastases are most frequently encountered in advanced PDAC [15]. In the last 15 years, the mechanisms by which miRNAs regulate metastasis have been researched, and there is evidence to suggest that these miRNAs, also known as metastamiRs, regulate tumor suppressor genes, oncogenes, metastasis genes, and the genes involved in the epithelial–mesenchymal transition [16]. For example, miR-146a seems to promote tumorigenesis and metastasis in PDAC, along with miR-21, which has been suggested as a prognostic marker for local invasion and lymph node metastasis [17]. Moreover, serum miR-210 levels can present normal values in healthy pancreatic tissue and chronic pancreatitis; lower levels can be associated with PDAC and are proven to offer a higher risk of metastasis and vascular invasion [18].

For this study, we compared the expression of 84 miRNAs previously associated with different types of cancer but not specifically with PDAC and its characteristics in 30 tumoral tissues from PDAC patients and 16 non-tumoral pancreatic tissue samples. Furthermore, the association between the expression of differentially expressed miRNAs and the tumoral characteristics, mainly metastasis presence and site, was evaluated.

## 2. Materials and Methods

### 2.1. Sample Collection

Thirty patients diagnosed with PDAC were enrolled in the study. The tumoral pancreatic tissue was obtained via surgical pancreatic resection (*n* = 6) or with a 22-gauge needle during ultrasound endoscopy and fine needle aspiration (FNA) intended for standard cytologic examination (*n* = 24). Sixteen samples of non-tumoral pancreatic tissues were obtained from the peritumoral area from patients who underwent surgical resection for a PDAC (*n* = 8), a neuroendocrine tumor (*n* = 4), an intraductal papillary mucinous neoplasm (*n* = 2), an adenosquamous carcinoma (*n* = 1), and metastasis of colorectal carcinoma (*n* = 1). The absence of tumoral cells in the non-tumoral pancreatic tissue was confirmed by a pathologist. In order to preserve the RNAs from degradation, the samples were collected and immersed in RNAs later for 48–72 h. The samples were stored in the Victor Babes National Institute of Pathology biobank at −80 °C until total RNA isolation. The study was approved by the ethics committees of the two institutions involved in the study: the Clinical Emergency Hospital of Bucharest (approval number 1960 of 28 February 2019), where the patients were recruited and the samples were collected, and the Victor Babes National Institute of Pathology (approval number 78 of 3 December 2019), where the experiments were performed. The samples included in the study were collected from 31 March 2019 to 20 April 2022. The miRNA expression analysis, performed at the Victor Babes National Institute of Pathology, started in February 2020. All individuals signed an informed consent to participate in the study and agreed to the use of their biological samples in accordance with the Declaration of Helsinki.

### 2.2. Evaluation of miRNAs

A spectrophotometric method (NanoDrop 2000, Thermo Scientific, Wilmington, NC, USA) was used to evaluate the quality and quantity of the total RNA isolated using the miRNeasy Mini Kit (Qiagen, Hilden, Germany). Ten ng of total RNA were reverse transcribed with an miRCURY LNA RT Kit (Qiagen, Hilden, Germany), and the expression of 84 miRNAs was evaluated using miRCURY LNA miRNA Focus PCR Panel Human Cancer YAHS-102 (Supplementary Table S1) and the miRCURY LNA SYBR Green PCR Kit (Qiagen, Hilden, Germany). The Ct values were screened in order to determine the miRNAs not expressed in the pancreatic tissue (tumoral and non-tumoral). miRNAs showing Ct values > 35 were excluded from the analysis. Supplementary Table S2 shows the Min-Max Ct values for each miRNA. The geometric mean of two reference mRNAs SNORD38B and SNORD49A was used to normalize the Ct data of the miRNAs of interest. The stability of SNORD38B and SNORD49A in non-tumoral pancreatic and PDAC tissues was verified using the RefFinder algorithm, analyzing three candidate reference RNAs (housekeeping genes) [19]. The miRNA expression data are reported as 2^−∆Ct^ values and in terms of Fold Change (FC). The 2^−ΔCT^ mean values and the fold change (FC = 2^−ΔΔCT^) were calculated as previously described by Livak et al. [20]. The FC values were calculated using different groups as references and according to the following comparisons: (i) FC (Tumoral vs. CTRL) = 2^−ΔCt^ T/2^−ΔCt^ CTRL; (ii) FC (PDAC HM vs. PDAC no-HM) = 2^−ΔCt^ HM/2^−ΔCt^ no-HM.

### 2.3. Statistical Analysis

**Figure 1:**
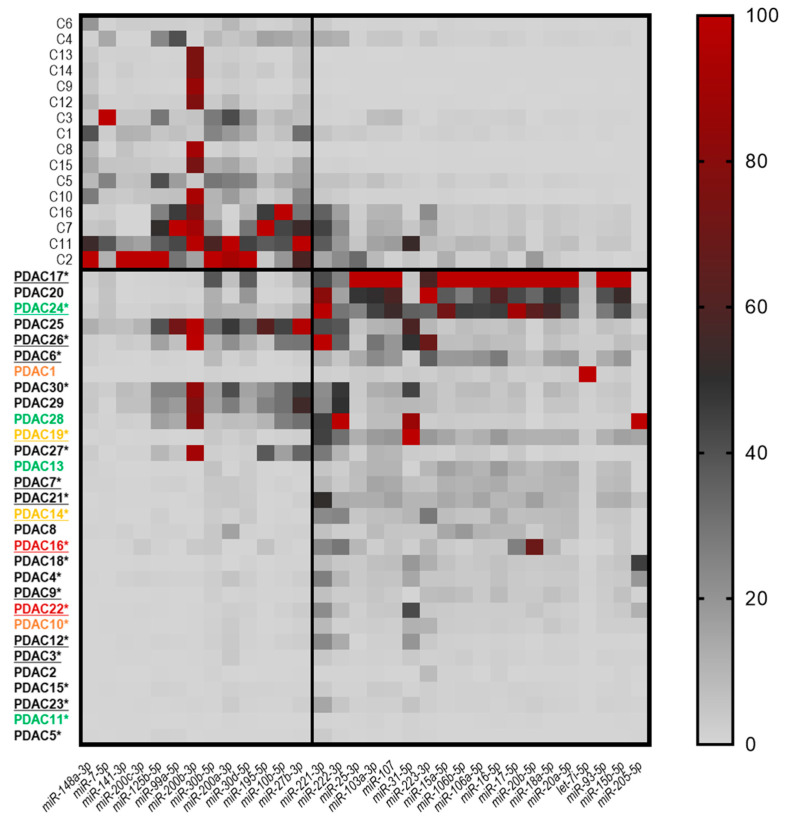
Heat map representing the expression of the 32 miRNAs found to be dysregulated in PDAC vs. CTRL tissues with *p* < 0.05 and 0.5 ≥ FC ≥ 2. Red indicates upregulation, light gray indicates downregulation, and dark gray indicates unmodified miRNA expression. After transforming the 2^−ΔCt^ values, the miRNAs were scaled in a manner that considered the lowest value as 0% and the highest value as 100% and ordered according to their expression levels. The columns refers to the miRNA symbols, and the rows refer to individual tissue samples. The color of the patient code reported in the rows indicates anatomic location (black = head; green = neck, body = red, body–tail = orange, and tail = yellow). The underlined codes refer to patients reporting hepatic metastasis, and * indicates the patients with lymph node involvement.

The Shapiro–Wilk test was performed on miRNA expression data to test the normality of the distribution. Since the miRNA levels were not normally distributed (*p* < 0.05), nonparametric tests were used for the analysis of data. The Mann–Whitney test was performed to compare the miRNA expression between the PDAC and CTRL groups, as well as between PDAC HM vs. PDAC no-HM, CTRL vs. PDAC HM, and CTRL vs. PDAC no-HM. The changes in miRNA levels were considered significant if *p* < 0.05 and 0.5 ≥ FC ≥ 2. A chi-squared test and Student’s *t*-test were used to compare the sociodemographic characteristics of the groups for categorical and continuous variables, respectively. The Statistical Package for the Social Sciences (SPSS version 17.0) was also used for statistical analysis. GraphPad Prism 8.4.3 was used to develop graphical representations of the results. The heat map reported in Figure 1 was generated using the 2^−ΔCt^ values of miRNAs with *p* < 0.05 and 0.5 ≥ FC ≥ 2. Data were transformed by using statistical software in order to obtain values from 0 to 100 and are graphically represented with different shades of colors. The expression of the significantly dysregulated miRNAs reported in Figure 1 was investigated in six similar studies found in dbDEMC (https://www.biosino.org/dbDEMC/index, accessed on 17 July 2023) in which the miRNA expression profile of PDAC tissue was compared with the non-tumoral pancreatic tissue. Reactome Pathway analysis via miRPathDB [21] was performed for each up- and downregulated miRNA list in the PDAC vs. CTRL comparison. Significant pathways targeted by a minimum 50% of the miRNAs in each list (10 for the upregulated miRNAs and 7 for the downregulated miRNAs) were reported. A receiver operating characteristic (ROC) curve was created and the area under the curve (AUC) was calculated by SPSS to assess the potential value of the selected the miRNAs discriminating patients with and without hepatic metastasis.

## 3. Results

This study included 30 cancerous tissues from PDAC patients and 16 non-tumoral pancreatic tissues from individuals who underwent pancreatic surgical resection for different reasons. The two groups showed no significant differences in terms of sex distribution (*p* = 0.216, χ^2^ = 1.533) or age (mean age PDAC = 61.23 ± 8.91; mean age CTRL = 56.18 ± 12.29, *p* = 0.117). The clinical data of PDAC patients are reported in Table 1.

In our initial analysis, we compared the expression levels of the 84 candidate miRNAs between the PDAC and non-tumoral tissues. Overall, 3 out of the 84 investigated miRNAs were not expressed in the pancreatic tissue, showing a Ct value above 35 both in the tumoral and non-tumoral tissue (miR-149-3p, miR-202-3p, and miR-206). The case–control analysis revealed 32 significantly dysregulated miRNAs (19 upregulated and 13 downregulated) within the tumoral tissue (Figure 1 and Supplementary Table S2). As reported in Figure 1, the five most upregulated miRNAs in the PDAC tissues were miR-205-5p (FC = 87.35; *p* = 0.006), miR-15b-5p (FC = 11.60; *p* < 0.001), miR-93-5p (FC = 8.84; *p* = 0.001), let-7i-5p (FC = 8.02; *p* = 0.01), and miR-20a-5p (FC = 7.59; *p* < 0.001), whereas the five most downregulated were miR-148a-3p (FC = 0.07; *p* < 0.001), miR-7-5p (FC = 0.10; *p* = 0.002), miR-141-3p (FC = 0.12; *p* = 0.003), miR-200c-3p (FC = 0.14; *p* = 0.0020, and miR-125b-5p (FC = 0.24; *p* = 0.001)

**Figure 2:**
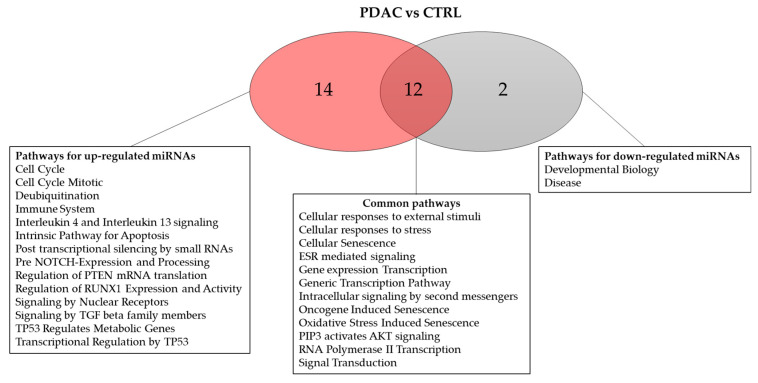
Number of Reactome pathways over-represented for miRNAs that were significantly dysregulated in PDAC tissues compared to the control tissues (results presented in Figure 1 and Supplementary Table S3).

We further compared our results on the 19 upregulated and 13 downregulated miRNAs with those obtained in other similar studies (PDAC tissue vs. non-tumoral pancreatic tissue, analyzed via microarray, RNA-seq, and gene expression array) identified using the dbDEMC database. The analysis revealed that 13 and 7 miRNAs were confirmed as being upregulated and downregulated, respectively, in PDAC tissue, whereas antithetical results were obtained for 3 miRNAs (Supplementary Table S3). As one pathway is typically regulated by several miRNAs and each miRNA is involved in several pathways, we detected any pathways regulated by the differentially expressed miRNAs in PDAC patients. Reactome pathway over-representation was performed for all of the differentially expressed miRNAs depicted in Figure 1. miRNAs that are significantly upregulated and downregulated in the PDAC group are involved in 26 and 14 pathways, respectively, and 12 of them are common (Figure 2).

**Figure 3:**
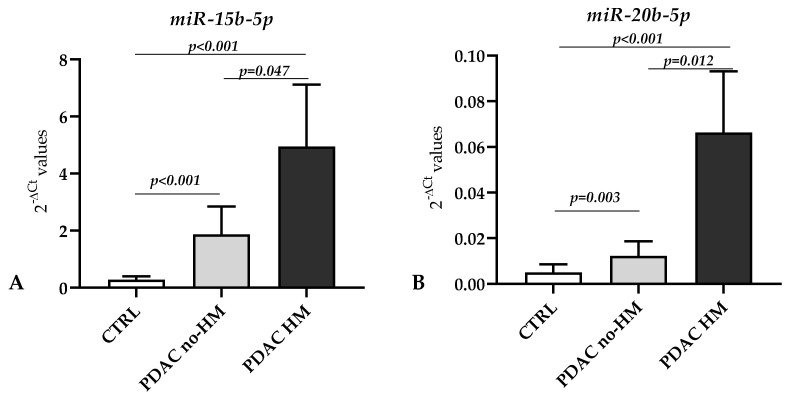
The two miRNAs found differentially expressed between PDAC HM and PDAC no-HM. (**A**) miR-15b-5p (FC = 2.65) and (**B**) miR-20b-5p (FC = 5.44). Bars indicate the mean of expression ± SEM. Statistical significance between the two PDAC groups and between CTRL vs. the two PDAC groups was calculated using the Mann–Whitney test.

**Figure 4:**
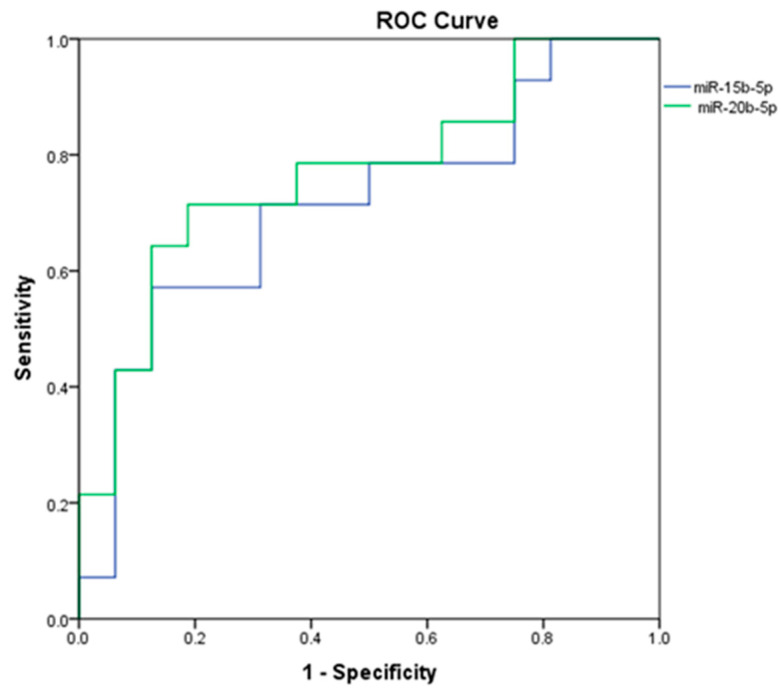
ROC curves for the two potential miRNA biomarkers (miR-15b-5p and miR-20b-5p) that seem to be able to discriminate between patients with/without hepatic metastasis.

We further analyzed any possible association between the levels of miRNAs found dysregulated in the case–control study and the presence of metastasis. Considering the comparison between tumoral tissues from patients with hepatic metastasis (PDAC HM) vs. those without hepatic metastasis (PDAC no-HM), we found an upregulation in the HM group of the following miRNAs: miR-15b-5p (FC = 2.65; *p* = 0.047) and miR-20b-5p (FC = 5.44; *p* = 0.012), as shown in Figure 3. The ROC curve assessing the potential value of miR-15b-5p and miR-20b-5p in discriminating the presence/absence of hepatic metastasis revealed an AUC = 0.714 (Sensitivity/1 − Specificity = 0.929/0.813, *p* = 0.046) for miR-15b-5p and AUC = 0.768 (Sensitivity/1 − Specificity = 0.929/0.750, *p* = 0.013) for miR-20b-5p (Figure 4).

No significant difference in miRNAs expression was observed in relation to anatomic location, lymphovascular invasion, lung metastasis, and the presence of diabetes (*p* > 0.05).

## 4. Discussion

In this study, the expression of 84 miRNAs involved in cancer was evaluated in 30 cancerous tissues from PDAC patients and 16 non-tumoral pancreatic tissues. A comparison between the tumoral and control tissues revealed that 32 miRNAs were significantly dysregulated in the PDAC group.

Reactome pathway over-representation analysis, performed separately on upregulated and downregulated miRNAs, revealed that these miRNAs are involved in 26 and 14 pathways, respectively, with 12 of them being common. The miRNAs in PDAC samples potentially target the following Reactome terms: “ESR-mediated signaling”, “PIP3 activates AKT signaling”, and “Regulation of PTEN”, among others.

Estrogen, acting through estrogen receptors, was found extensively dysregulated during the development and progression of different cancers [22,23,24], including pancreatic cancer, where the potential mechanisms of ESR signaling are still poorly understood [25].

In multiple malignancies such as pancreatic cancer, cytokines, chemokines, and growth factors activate the class I phosphoinositide 3-kinase (PI3K)/protein kinase B (AKT)/mammalian target of rapamycin (mTOR) (PI3K/AKT/mTOR) signaling pathway, which plays a fundamental role in motility regulation, metabolism, cell proliferation, cell migration, and cell survival [26]. Briefly, PI3K phosphorylates PIP2 to PIP3, leading to AKT activation, while PTEN, a lipid phosphatase, dephosphorylates PIP3 and is considered a negative regulator of this pathway. In pancreatic cancer, the overexpression of this pathway has been observed in concert with a decrease in PTEN expression in 25–70% of cases [27], and recently, many small-molecule anticancer agents have been developed to regulate autophagy and apoptosis associated with pancreatic cancer treatment targeting the PI3K/Akt/mTOR pathway [28].

Through ranking the identified dysregulated miRNAs of our study according to the FC, we found that miR-205-5p was the most upregulated miRNA, showing a FC of 83.35, whereas the most downregulated miRNA was miR-148a-3p, which showed a FC of 0.068.

Contrasting findings on miR-205-5p expression in PDAC have been described in the literature. In line with our results, Qin et al. observed the upregulation of miR-205-3p in PDAC cell lines and PDAC tissues compared to normal cell lines and tissues, respectively. Moreover, the authors showed that, by targeting APC, miR-205-3p could encourage cell proliferation in pancreatic cancer [29]. Other studies on human tissues and blood serum samples have revealed miR-205 overexpression in PDAC patients compared to healthy controls [30,31]. Additionally, an increase in the expression of miR-205 in the pancreatic juice collected from patients with PDAC compared with those without pancreatic illness was identified [32]. On the contrary, a significant downregulation in miR-205 in human pancreatic cancer cell lines and PDAC tissue compared to adjacent tissue has been observed by Zhuang et al. [33] and confirmed by other research groups [34,35,36,37]. In addition, Zhuang et al. showed that low miR-205 expression was associated with poor prognosis in patients with pancreatic cancer [33]. Notably, this miRNA has been found to target the PI3K/AKT signaling pathway in different malignancies [38,39,40].

Among the miRNAs found downregulated in our study in tumoral PDAC tissues, miR-148a-3p had the smallest fold change and a significance value of *p* < 0.001.

In various cancers, including PDAC, miR-148a-3p has been demonstrated to have low expression [41,42]. According to Hanoun et al., hypermethylation of the DNA region encoding miR-148a is an early event that occurs in preneoplastic pancreatic intraepithelial neoplasia (PanIN), which induces miR-148a transcriptional repression in both PanIN and PDAC samples [43]. Moreover, another research group showed that, in pancreatic cancer, miR-148a-3p inhibits the epithelial–mesenchymal transition and stemness properties via the Wnt1-mediated Wnt/-catenin pathway [44]. Again, in line with our results, Liffers et al. discovered that miR-148a is downregulated in human pancreatic ductal adenocarcinomas and that, by targeting CDC25B, it regulates cell survival [45]. This result was not confirmed in a study by Popov et al., which did not find differences in the expression levels of miR-148a between cancer and normal tissues but identified positive and negative correlations between the level of this miRNA with the tubular tumor growth pattern and the dissociated growth pattern and nuclear atypia, respectively [46].

In our study, we also found two miRNAs—miR-15b-5p and miR-20b-5p—that were both upregulated in the case–control study, showing a higher expression level in PDAC tissues from patients with hepatic metastasis compared with those not reporting metastasis. The first signs of an association between pancreatic cancer and miR-15b-5p was reported by Zhang et al. in a study published in 2009, which, in line with our findings, reported that miR-15b-5p was upregulated in pancreatic cancer tissues and cell lines compared to normal controls [47]. However, another study that integrated public databases reported antithetical results, suggesting the downregulation of this miRNA in pancreatic cancer [48]. Interestingly, the levels of miR-15b-5p in pancreatic cancer have been investigated in relation to SMURF2, a protein with a tumor-suppressive function. The authors found that miR-15b was upregulated in pancreatic cancer and promoted the epithelial–mesenchymal transition by degrading SMURF2 [49].

Regarding the role of miR-20b-5p in pancreatic cancer, so far, only one study has investigated this malignancy, and most of the reported results are related to the involvement of this miRNA in hepatocellular carcinoma (HCC). Integrated data mining analysis identified the PVT1/miR-20b/CCND1 axis as a promising pathway-related ceRNA axis in the progression of pancreatic cancer [50]. Regarding the involvement of this miRNA in HCC, it has been found that the overexpression of miR-20b-5p facilitates proliferation, migration, and invasion and suppresses the apoptosis of malignant cells [51] and that LncRNA WWOX-AS1 sponges miR-20b-5p in HCC, repressing its progression by upregulating WWOX [52].

## 5. Conclusions

The current study identified a panel of miRNAs differentially expressed in cancerous tissues from PDAC patients and non-tumoral pancreatic tissues and listed the signaling pathways underlying this malignancy. These miRNAs and pathways can be considered as potential targets for new treatment strategies. Moreover, for the first time in the literature, our study identified miR-15b-5p and miR-20b-5p as potential markers of hepatic metastasis in PDAC. One of the limitations of this study is the relatively small cohort analyzed, which is partially due to the invasive procedure needed to obtain the tumoral tissue. In order to overcome this limitation, we are planning to extend the study by collecting other PDAC tissues and the blood of PDAC patients and analyzing the circulating miRNAs. This will allow us to validate our results in peripheral and more accessible tissues and enable the identification of new miRNAs as prognostic and predictive biomarkers in PDAC in a large cohort of patients.

## Figures and Tables

**Table 1:** Clinical data of PDAC patients involved in the study.

PDAC (*n* = 30)	
Anatomic Location	Head = 20
	Neck = 4
	Body = 2
	Body-Tail = 2
	Tail = 2
Stage (UICC) IA/IB/IIA/IIB/III/IV	1/4/4/1/6/14
Lymph Node Involvement	Yes = 22 (73.3%)
	No = 8 (26.7%)
Hepatic Metastasis	Yes = 14 (46.7%)
	No = 16 (53.3%)
Lung Metastasis	Yes = 2 (6.7%)
	No = 28 (93.3%)
Diabetes	Yes = 12
	No = 18
Smokers	Yes = 15
	No = 12
	Not available data = 3
Alcohol consumers	Yes = 4
	No = 23
	Not available data = 3
Vegetarian diet	Yes = 1
	No = 29

## Data Availability

Data are contained within this article and the Supplementary Materials. Raw data can be obtained from the corresponding author upon reasonable request.

## Supplementary Materials

The following supporting information can be downloaded at: https://www.mdpi.com/article/10.3390/genes14081577/s1, Table S1: Description of the Human Cancer YAHS-102 panel reporting the analyzed miRNAs, Table S2: Ct range of the analyzed miRNAs in pancreatic tissue (tumoral and non-tumoral), Table S3: Significantly dysregulated miRNAs in the PDAC vs. CTRL comparison.
