# Supplementary material for: Increased Levels of miR-15b-5p and miR-20b-5p in Pancreatic Ductal Adenocarcinoma with Hepatic Metastases
Source: Genes (Basel). 2023 Aug 2;14(8):1577. doi: 10.3390/genes14081577 (PMC10454474; doi:10.3390/genes14081577)
Supplement: Supplementary file 1 [file genes-14-01577-s001.zip › Supplementary Table S3.pdf]

Supplementary Table S3: Significantly dysregulated miRNAs in the comparison PDAC vs CTRL in our study and results obtained in similar studies reported in dbDEMC (https://www.biosino.org/dbDEMC/index)

	**Ou results**		**dbDEMC EXP00134**	**dbDEMC EXP00137**	**dbDEMC EXP00248**	**dbDEMC EXP00249**	**dbDEMC EXP00416**	**dbDEMC EXP00595**
**miRNA**	**FC***	**p-value#**	**5PDAC vs 4 NT**	**25 PDAC vs 7 NT**	**9 PDAC vs 9 NT**	**7 PDAC vs 5 NT**	**178 PDAC vs 4 NT**	**12 PDAC vs 3 NT**
miR-205-5p	87.35	0.006						
miR-15b-5p	11.6	<0.001	Up					
miR-93-5p	8.84	0.001				Up		
let-7i-5p	8.02	0.01			Up			
miR-20a-5p	7.59	<0.001						
miR-18a-5p	7.55	<0.001	Up					
miR-20b-5p	7.42	<0.001						
miR-17-5p	7.12	<0.001	Up			Up		
miR-16-5p	6.96	<0.001						
miR-106a-5p	6.1	0.001						
miR-106b-5p	6.09	<0.001	Up			Up		Up
miR-15a-5p	4.78	0.004	Up					
miR-223-3p	4.61	<0.001			Up			
miR-31-5p	4.09	0.001			Up			Up
miR-107	3.53	0.011	Up		Up			
miR-103a-3p	3.1	0.012						
miR-25-3p	2.66	0.015						
miR-222-3p	2.52	0.02	Up	Up	Up			Up
miR-221-3p	2.46	0.009			Up			Up
miR-210-3p	1.6	0.003	Up			Up		Up
miR-27b-3p	0.5	0.001						Down

miR-10b-5p	0.42	0.03			Up		Down	Up
miR-195-5p	0.41	0.004						
miR-30d-5p	0.35	0.018						
miR-200a-3p	0.34	0.014	Up	Up			Up	
miR-30b-5p	0.34	0.03						Down
miR-200b-3p	0.34	<0.001		Up			Up	
miR-99a-5p	0.28	0.002	Down					Up
miR-125b-5p	0.24	0.001	Down		Up			Up
miR-200c-3p	0.14	0.002	Up	Up			Up	
miR-141-3p	0.12	0.003						Down
miR-7-5p	0.1	0.002						
miR-148a-3p	0.07	<0.001	Down			Down		Down

* FC= Fold change

# p-value= Mann Whitney test

Up= adj-p<0.05 and FC≥2

Down= adj-p<0.05 and FC≤0.5
